# One-Year Analysis of Risk Factors Associated With Cognitive Impairment in Newly Diagnosed Epilepsy in Adults

**DOI:** 10.3389/fneur.2020.594164

**Published:** 2020-11-06

**Authors:** Nan Li, Jing Li, Yanyan Chen, Chaojia Chu, Xin Zhang, Rui Zhong, Mengmeng Li, Yingxue Lu, Qian Zhao, Weihong Lin

**Affiliations:** ^1^Department of Neurology, The First Hospital of Jilin University, Changchun, China; ^2^Department of Neuroelectrophysiology, Changchun Six Hospital, Changchun, China

**Keywords:** adult, anti-seizure medication, cognitive impairment, newly diagnosed epilepsy, risk factor

## Abstract

Cognitive impairment (CI) occurs in people with epilepsy, affecting their quality of life. This study aimed to identify factors associated with CI in adult patients with newly diagnosed epilepsy. Additionally, we sought to determine whether any particular cognitive function is impaired predominantly by anti-seizure medications or by other factors. We enrolled 229 patients with newly diagnosed epilepsy and 191 participants were followed up for 1 y. We used the Montreal Cognitive Assessment as a tool to quantify CI. The sub-item scores were also collected to assess whether any aspects of CI are predominantly affected by anti-seizure medication treatment. Subjective memory decline due to anti-seizure medications was also recorded. One-hundred-and-two participants (44.5%) had CI onset before anti-seizure medication treatment. Aging, low education level, stroke or brain surgery etiology, and anxious symptoms were identified as risk factors for CI before anti-seizure medications use. Brain surgery for the young, anxious status for the middle-aged, and depressive status for the elderly were risk factors for CI at different ages. The elderly PWE had worse memory than the others. PWE with TLE had worse cognition, especially in memory and naming. The overall impact of anti-seizure medications on cognition was mild. Refractory epilepsy was a predictor of cognitive decline. Subjective memory decline was predicted by high-risk treatment and by a finding of refractory epilepsy. Clarifying the risk factors for CI can help the physician to assess the probable risk of CI for each individual before the start of anti-seizure medication treatment, which may lead to better compliance.

## Introduction

More than 70 million people worldwide have epilepsy (1). Many people with epilepsy (PWE) experience cognitive impairment (CI), and 30% of PWE have interictal disturbances of memory and attention (2). CI is the biggest factor influencing the quality of life of PWE (3). Consequently, it is necessary to identify factors associated with CI in PWE before any anti-epileptic treatment and to establish whether a decline in cognitive function is related to anti-seizure medication treatment or other factors. There is a need for data on CI in adult PWE, but existing studies have focused primarily on pediatric patients with epilepsy (4, 5). Studies of predictors of cognitive comorbidity in adult PWE have mostly focused on specific types of epilepsy, such as temporal lobe epilepsy (6) or post-surgical epilepsy (7).

Multiple factors influence cognition. Previous studies have identified the underlying etiology and the anatomical location of the epileptic foci as factors influencing cognition (8, 9). In addition, mood disorder, a common comorbidity of epilepsy, is also an important factor in CI (10), because CI is a core feature of depression (11). Aging also leads to cognitive decline (12, 13). These factors should all be considered when assessing the impact of anti-seizure medications and other factors on CI in PWE. Nevertheless, reports on new-onset adult PWE are rare.

This study sought to identify factors associated with CI in adult patients with newly diagnosed epilepsy (NDE). Furthermore, we attempted to identify whether any particular cognitive function is impacted predominantly by anti-seizure medications or by other factors. In this study, we used the Montreal Cognitive Assessment (MoCA) as a tool to quantify CI, because it is more sensitive than the Mini-Mental State Examination and has been used in many studies to detect mild cognitive impairment (14). The sub-item scores were also collected to determine the aspects of CI predominantly influenced by anti-seizure medication treatment. Subjective memory decline due to anti-seizure medications was also recorded and analyzed.

## Materials and Methods

### Patient Recruitment

Patients visiting the Epilepsy Diagnosis and Treatment Center of the First Hospital of Jilin University who were newly diagnosed with epilepsy and prescribed anti-seizure medications were enrolled in this experiment between September 2016 and 2018 and followed up until September 2019.

The definition of epilepsy we used conforms to the practical clinical definition published by the International League Against Epilepsy (ILAE) in 2014 (15), and our classification of seizure met the diagnostic criteria published by the ILAE in 2017 (16). The 1989 ILAE classification (17) was applied to categorize epileptic syndrome. We diagnosed refractory epilepsy according to the new definition published in 2010 (18). The definition of a patient with NDE used in this study was a person with confirmed epilepsy who had not been diagnosed specifically with epilepsy or treated with anti-seizure medication previously. Before being diagnosed, all participants underwent a thorough clinical and laboratory investigation, including a 24 h video electroencephalogram (EEG) and 3.0-T high-resolution brain magnetic resonance imaging (MRI).

The inclusion criteria were: (1) met the diagnostic criteria for epilepsy; (2) had not received antiepileptic treatment previously; (3) could complete the assessment scales independently; (4) signed an informed consent form for participation; (5) more than 16 y old.

The exclusion criteria were: (1) status epilepticus only; (2) progressive neurologic disorder; (3) psychotic disorder; or (4) audio-visual dysfunction.

### Study Procedure

At their first visit, the patients were diagnosed by a professional neurologist and were administered an anti-seizure medication following the 2012 guidelines of the National Institute for Health and Clinical Excellence (19). For patients who met the inclusion criteria, a baseline file was completed, which contained demographic, symptomatic, aetiologic, and nervous-system data, as well as the results of a systematic physical examination, a laboratory examination, an EEG, and an MRI. The demographic data were collected by interview. Participants were then asked to complete a series of scales, including the MoCA, the Generalized Anxiety Disorder 7-item Scale (GAD-7), and the Chinese version of the Neurological Disorders Depression Inventory for Epilepsy (c-NDDI-E), to estimate their cognitive function and mood. The MoCA total scale and its sub-item scores, including visuospatial and executive function, naming, attention, calculation, story-retelling fluency, abstract thinking, memory, and orientation, were recorded. The cut-off point for the total MoCA score was education-based: it was normally set to 26, but one point was added for participants with educational attainment less than senior high school.

The participants were instructed to visit the hospital for treatment adjustments 1, 3, 6, and 12 months after diagnosis and in cases of seizure recurrence. At the 12-month time point, a follow-up file was completed for all patients, which recorded the patients' seizure types and frequency, the doses of the anti-seizure medications administered, any adverse effects, and the results of blood tests. The scales referred to above were also re-administered for the follow-up file. Subjective memory decline caused by anti-seizure medications was recorded on a 4-point scale (1, no symptoms; 2, mild symptoms; 3, moderate symptoms; and 4, severe symptoms), and the participants were asked repeatedly to confirm that the symptoms emerged as the treatment began.

### Statistical Analyses

#### Descriptive Analyses

The data of all participants included in the experiment were analyzed in this part, and a multiple linear regression model was used to identify factors associated with CI. Multivariate linear regression is suitable for confounding factor analysis. When analyzing a single variable, it controls for other variables. The MoCA total score was defined as the dependent variable, and age, age of onset, sex, marital status, education level, GAD-7 score, diagnosis of the epileptic syndrome, and etiology were analyzed as independent variables. Covariates that did not change the *B*-value of the MoCA by at least 10% were excluded from further analysis.

To identify characteristics that might lead to drop-out, chi-square tests and two-sample Mann–Whitney *U*-tests were performed to compare participants who completed the 12-month follow-up with those who were lost to follow-up.

#### Longitudinal Analyses

Only participants who completed the last follow-up visit were included in the long-term analyses. Paired Wilcoxon signed-ranks tests were used to compare the MoCA total scores and its sub-items at the two-time points. Changes in the MoCA scores and sub-items, the GAD-7, the c-NDDI-E, and the seizure frequency were derived by calculating the differences between variables at two-time points (follow-up and baseline) for each individual. A high-risk condition was defined as polytherapy with three types of anti-seizure medications, where two had a defined daily dose (20), or treatment with topiramate. All other treatments were defined as low-risk (21). Multiple linear regression models were used to analyze statistically significant changes in the scores on the MoCA sub-items (dependent variables). Treatment-risk conditions present at follow-up and changes in the GAD-7 score were defined as further dependent variables for each individual. The significant variables identified in the descriptive analysis were adjusted in this model. The logistic regression model was also used to analyze the factors influencing the presence of some degree of subjective memory impairment (i.e., mild to severe levels). Subgroup analysis were applied due to the heterogeneity of the participants in terms of age, epileptic syndrome, and refractory epilepsy. The participants were divided into the young group (age < 45), the middle-aged group (45 ≤ age < 59), and the elderly group (age ≥ 60).

Values for continuous variables are expressed as mean ± standard deviation (SD), and values for categorical variables are expressed as frequencies (%). All *p*-values were from two-tailed tests. *p* < 0.05 was considered to indicate statistical significance. The data were inputted by EpiData software (The EpiData Association, Odense, Denmark) and were subsequently analyzed using SPSS for Windows, Version 16.0 (SPSS Inc., Chicago, IL, USA).

### Ethical Approval

The protocol for this study was approved by the Ethics Committee of the First Hospital of Jilin University and was performed in accordance with the ethical standards laid down in the 1964 Declaration of Helsinki and its later amendments. Each enrolled patient provided a signed informed consent form before the study start.

## Results

**Figure 1:**
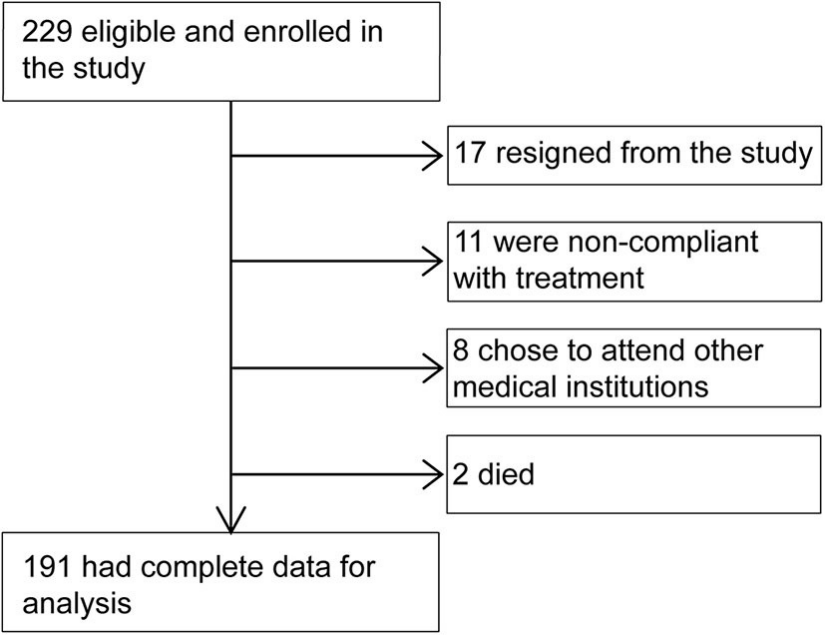
Participant flow chart.

A total of 229 patients were enrolled in the study. During follow-up, 36 patients dropped out (17 resigned from the study, 11 were non-compliant with treatment, and 8 chose to attend other medical institutions), and 2 died (1 of stroke and 1 of unknown cause). Eventually, 191 participants completed the 12-month follow-up. The study flowchart is shown in Figure 1.

### Baseline Characteristics

**Figure 2:**
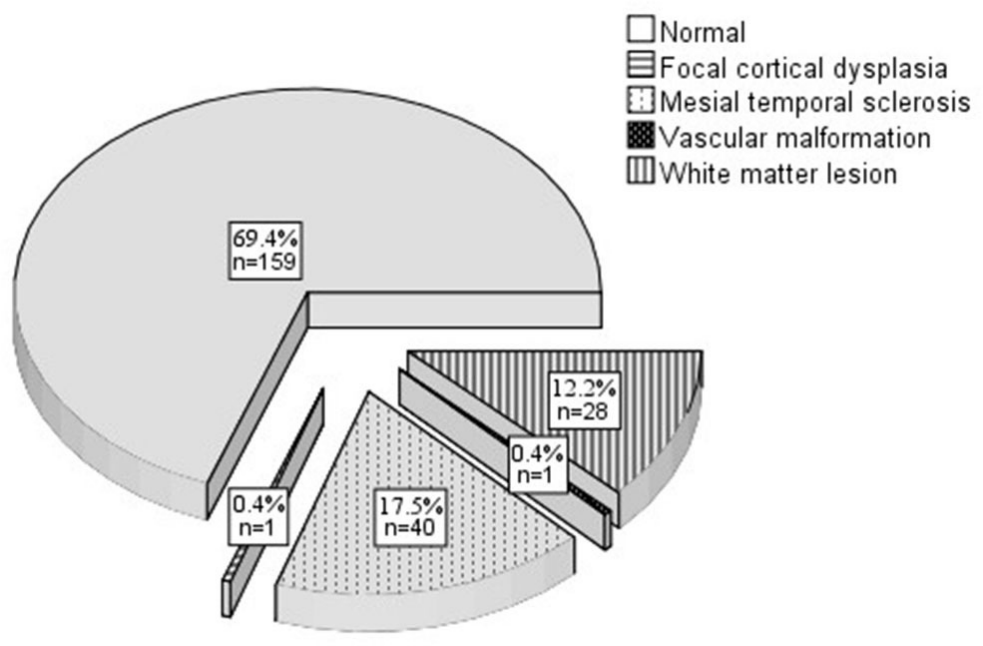
MRI finding at baseline.

The baseline characteristics of the participants are summarized in Table 1. A wide age range was covered (16–86 y; 38.2 ± 16.4 y). Most of the study population was male and married or partnered (60.3%, *n* = 138). Most participants were educated beyond primary school. The majority were employed and had an average income of more than 140 US dollars per month (86.9%, *n* = 199). No participant exhibited tobacco or alcohol abuse/dependence and most of our participants did not smoke or drink currently. Nine participants (3.9%) had an abnormal birth history (3 premature birth, 5 intrauterine asphyxia, 1 birth injury) and 25 (10.9%) had a history of febrile convulsion (19 simple type, 6 complex types). Twenty-seven participants (11.8%) had a family history of epilepsy. Other family history included migraine (6.1%, *n* = 14), psychotic disorder (4.8%, *n* = 11), and others (0.9%, *n* = 2). Seventy participants had an abnormal MRI (Figure 2). Ictal focal seizures were recorded in 32 participants (18 in the temporal lobe, 7 in the frontal lobe, 2 in the parietal lobe, 1 in the occipital lobe, 1 in the center, and 3 with unknown origin). The interictal epileptic discharge could be seen in most participants (89.1%, *n* = 204). Temporal lobe epilepsy (TLE) (27.9%, *n* = 64) and frontal lobe epilepsy (FLE) (10.5%, *n* = 24) were the most common epileptic syndromes. Almost half of the participants (44.5%; *n* = 102) had CI onset prior to starting an anti-seizure medication treatment. We found no statistically significant difference between participants who were included in the analysis and those lost to follow-up.

**Table 1:** Participant demographic characteristics.

**Variable**	**Included in the analysis (*n* = 191)**	**Lost to follow-up (*n* = 38)**	**Total at start (*n* = 229)**
Age, y	37.1 ± 15.7	43.7 ± 19.0	38.2 ± 16.4
Sex, *N* (%)			
Male	120 (62.8)	20(52.6)	140 (61.1)
Female	71 (37.2)	18(47.4)	89 (38.9)
Formal educational attainment, *N* (%)			
No formal or primary school	15 (7.9)	6 (15.8)	21 (9.2)
Junior high school	90 (47.1)	12 (31.6)	102 (44.5)
Senior high school	27 (14.1)	11 (28.9)	38 (16.6)
Tertiary	59 (30.9)	9 (23.7)	68 (29.7)
History of smoking, *N* (%)			
Current	40 (20.9)	6 (15.8)	46 (20.1)
Previous	22 (11.5)	28(21.1)	30 (13.1)
Never	129 (67.5)	24 (63.2)	153 (66.8)
History of alcohol use, *N* (%)			
Current	37 (19.4)	6 (16.7)	43 (18.8)
Previous	46 (24.1)	9 (25.0)	55 (24.0)
Never	108 (56.5)	23 (60.5)	131 (57.2)
Duration of disease, y	3.8 ± 7.2	3.5 ± 5.4	3.7 ± 7.0
Seizure frequency, per month	4.5 ± 8.5	4.2 ± 7.6	4.4 ± 8.4
MoCA^a^	24.5 ± 4.7	23.4 ± 5.3	24.4 ± 4.8
GAD-7^b^	4.3 ± 4.2	5.7 ± 5.6	4.6 ± 4.5
c-NDDI-E^c^	8.5 ± 3.2	9.6 ± 4.6	8.7 ± 3.5

*Values are mean (standard deviation) or mean ± 95% CI*.

a *MoCA: Montreal cognitive assessment*.

b *GAD-7: Generalized Anxiety Disorder 7-item Scale*.

c *c-NDDI-E: c-NDDI-E, the Chinese version of the Neurological Disorders Depression Inventory for Epilepsy*.

Multiple linear regression analyses were used to model the association between the MoCA total scores and baseline factors (*R*^2^ = 0.52). The statistically significant results are presented in Table 2. Aging was the predominant factor leading to a decline in objectively assessed cognitive function (Beta = −0.45). Taking tertiary education as a reference, participants with no formal or primary education had a poorer cognitive function. The sample size of those who were widowed was too small to allow a decision as to whether this factor influenced CI in PWE. Stroke and brain surgery were predictors of CI, compared with uncertain etiology. The GAD-7 scores had a negative correlation with the MoCA scores; moderate drinking and higher-income had positive correlations.

**Table 2:** Results of the multiple linear regression model for baseline factors influencing MoCA (*n* = 229).

**Variable**	**B**	**Beta**	***p*-value**
Age, y	−0.13	−0.45	<0.001
Average income, USD/mon	0.58	0.15	0.007
GAD-7^a^	−0.18	−0.17	0.002
Formal educational attainment			
No formal or primary school	−4.52	−0.27	<0.001
Tertiary		Reference	
History of alcohol use			
Current	1.57	0.13	0.039
Never		Reference	
Etiology			
Stroke	−3.78	−0.15	0.004
Brain surgery	−3.62	−0.17	0.001
Uncertain		Reference	

a *GAD-7: Generalized Anxiety Disorder 7-item Scale*.

### Longitudinal Changes in Cognition

#### Clinical Characteristics

The clinical characteristics of the participants at baseline and at the 12-month follow-up are compared in Table 3. At the 12-month follow-up, 31.9% (*n* = 61) of the participants had become seizure-free, whereas 11.0% (*n* = 21) were diagnosed as having refractory epilepsy. Two participants withdrew from using anti-seizure medications because of objective adverse effects at 1 week before the 12-month follow-up, and we estimated their scales as planned. After a 12-month period of anti-seizure medication treatment, the seizure frequency had decreased (*Z* = −8.093, *p* < 0.001).

**Table 3:** Participant clinical characteristics (*n* = 191).

**Variable**	**At baseline**	**At follow-up**	***p*-value**
Anti-seizure medications, *N* (%)			
High-risk treatment		30 (15.7)	
Low-risk treatment		161 (84.3)	
Seizure frequency, per month, *N* (%)	4.5 ± 8.5	1.0 ± 3.7	<0.001
MoCA scale^a^			
Total	24.5 ± 4.8	24.5 ± 4.5	0.764
Visuospatial and executive	4.0 ± 1.3	3.8 ± 1.3	0.018
Naming	2.7 ± 0.6	2.7 ± 0.7	0.809
Attention	2.7 ± 0.5	2.7 ± 0.6	0.209
Calculation	2.7 ± 0.8	2.7 ± 0.7	0.347
Story-retelling fluency	2.3 ± 1.0	2.3 ± 1.0	0.414
Abstract thinking	1.5 ± 0.7	1.5 ± 0.7	0.781
Memory	2.3 ± 1.6	2.6 ± 1.6	0.014
Orientation	5.8 ± 0.7	5.8 ± 0.6	0.218
GAD-7^b^	4.3 ± 4.2	3.1 ± 3.8	<0.001
c-NDDI-E^c^	8.5 ± 3.2	7.8 ± 2.8	0.006
Subjective memory decline			
None		126 (66.0)	
Mild		47 (24.6)	
Moderate		14 (7.3)	
Severe		4 (2.1)	

*Values are mean (standard deviation) or mean ± 95% CI*.

a *MoCA: Montreal cognitive assessment*.

b *GAD-7: Generalized Anxiety Disorder 7-item Scale*.

c *c-NDDI-E: c-NDDI-E, the Chinese version of the Neurological Disorders Depression Inventory for Epilepsy*.

#### Overall Changes in Cognition

When we compared the baseline and 12-month follow-up data, we found no significant difference in the MoCA total scores. However, the sub-item scores showed some differences. The scores for visuospatial and executive function (*Z* = 2.363, *p* = 0.018) and the memory scores (*Z* = 2.466, *p* = 0.014) had increased. And the scores of GAD-7 (*Z* = −4.601, *p* < 0.001) and c-NEED-E (*Z* = −2.743, *p* = 0.006) both decreased. When comparing the MoCA total scores at baseline and at follow-up in participants with refractory epilepsy vs. those that became seizure-free, the latter performed better at the 1-y time point (*Z* = −1.976, *p* = 0.048). Seventeen participants (8.9%) had subjective memory decline.

The use of high-risk treatments, changes in the GAD-7 scores, and variables found significant in the previous multiple linear regression analysis were analyzed as independent variables in the assessment of longitudinal changes in cognition. The covariates that did not change the *B*-value of sub-items by at least 10% were excluded from the models. Increases in GAD-7 (Beta = −0.23) and use of high-risk treatments (Beta = −0.19) negatively correlated with visuospatial and executive function. No memory-correlated factor was found. Changes in the c-NDDI-E score were adjusted in a later model, but this did not change the results. The results of multiple linear regression modeling of the sub-items are presented in Table 4.

**Table 4:** Results of multiple linear regression analysis for factors influencing cognitive function (*n* = 191).

**Model input**	**Visuospatial and executive (*****R***^****2****^ **=** **0.136)**		**Memory (*****R***^****2****^ **=** **0.059)**	
	***B***	***p*-value**	***B***	***p*-value**
Change in GAD-7^a^	−0.06	0.002	Excluded	
Treatment-risk conditions	−0.53	0.010		

a *GAD-7: Generalized Anxiety Disorder 7-item Scale*.

We included subjective memory decline in the logistic regression analysis; treatment-risk conditions were the only factors remaining in the model (OR = 5.03, 95% CI = 1.58–16.00, *p* = 0.006). When we stratified the low-risk group by type of anti-seizure medication therapy (monotherapy vs. two-drug therapy), we found no significant difference between these groups in terms of subjective memory decline. Subjective memory decline in patients that became seizure-free was less than in those with refractory epilepsy (*Z* = −2.013, *p* = 0.044).

#### Age-Subgroups' Changes in Cognition

Comparing the cognitive performance in the age-subgroups at baseline, the young group had the highest scores in MoCA total scores and visuospatial and executive function, naming and memory, and the elderly group had the lowest sub-item scores in memory. There was no significant difference between the middle-aged group and the elderly group in the MoCA total score. At the 12-month follow-up, the young group had the highest scores in mention and the MoCA total scale and sub-items mentioned before than the other two groups, and the elderly group had the lowest scores in memory as before. No significant difference was found in subjective memory decline between them.

For the young group, brain surgery was the predominant factor leading to a decline in the MoCA total score at baseline (Beta = −0.31, B = −4.96, *p* < 0.001), and moderate drinking and higher income were positive factors for improving MoCA score. Memory was the only sub-item that changed and it improved after 1 y of treatment (*Z* = −3.015, *p* = 0.003), but the use of high-risk treatments was the negative influencing factor (Beta = −0.22, B = −0.97, *p* = 0.013). For the middle-aged group, a higher GAD-7 score was the only factor leading to declining in the MoCA total score at baseline (Beta = −0.44, B = −0.59, *p* = 0.014). After 1 y of treatment, visuospatial and executive function improved (*Z* = −2.309, *p* = 0.021) and increased GAD-7 score was the only but mild negative factor (Beta = −0.38, B = −0.09, *p* = 0.038). For the elderly group, a higher c-NDDI-E score was the only influencing factor leading to a decline in the MoCA total score at baseline (Beta = −0.44, B = −0.63, *p* = 0.040). No sub-item changed after treatment. Age was adjusted in the multiple linear regression analysis but this did not change the results.

#### Epileptic Syndromes' Changes in Cognition

When we compared the cognitive performance between the two most common epileptic syndromes at baseline, the FLE group had higher scores in the MoCA total score (*Z* = −2.274, *p* = 0.023) and memory (*Z* = −2.430, *p* = 0.015) than the TLE group. After 1 y of treatment, the FLE group had better performance in naming (*Z* = −2.469, *p* = 0.014) and memory (*Z* = −2.600, *p* = 0.009) than the TLE group but the MoCA total scores did not show significant difference. The sample sizes of other epileptic syndromes were <5 so we excluded them from the analysis in this part.

Aging (Beta = −0.56, B = −0.17, *p* = 0.002), higher c-NDDI-E score (Beta = −0.41, B = −0.57, *p* = 0.001) and brain surgery (Beta = −0.31, B = −8.32, *p* = 0.012) were negative factors leading to lower MoCA total score at baseline in the TLE group, and no factor was found in the FLE group. Naming improved (*Z* = −2.000, *p* = 0.046) in the FLE group and higher GAD-7 score at the 12-month follow-up was the only but mild positive influencing factor (Beta = −0.45, B = −0.04, *p* = 0.036). There is no significant difference between the two groups in subjective memory decline.

## Discussion

Since few short-term, follow-up prospective studies focused on cognitive function in adults with NDE have been published, we explored the risk factors for CI in PWE before anti-seizure medication treatment and evaluated whether anti-seizure medication treatment influenced cognitive function. In this prospective study, we enrolled NDE patients and followed them for 1 y to distinguish the effect of anti-seizure medications from those of the various other factors contributing to CI. Furthermore, we included patients with almost all anti-seizure medication treatments available in China indiscriminately, to obtain a more representative sample.

### Risk Factors for CI in NDE

Almost half of the participants (44.5%) had CI at baseline, which was similar to the value (43%) reported in the review by Arjune et al. (22). We found a decline in objective cognition in NDE patients dependent on age, level of formal education, anxiety, and some etiologies, such as stroke and brain surgery. Moderate drinking and higher income predicted less CI.

The elderly adults had worse cognition in our study than the other PWE, especially in memory. Cognitive decline with aging is a natural phenomenon. For elderly adults, cognitive performance declines because of anatomical changes such as volume loss in the pre-frontal cortex and hippocampus, changes in gene expression, changes in physical neural characteristics, and the accumulation of numerous small infarcts (23). Elderly PWE has greater deficits in visual and verbal memory, executive function, attention, and psychomotor or processing speed due to ischemic stroke, neurological comorbidities, and higher body mass index compared with healthy elderly adults (22). Moreover, 25 y research reported that cognition declined faster in people with late-onset epilepsy (24). There is a significant intersection between Alzheimer's disease and late-onset epilepsy with unknown origin. Amyloid-β, the biomarker of Alzheimer's disease which might drive CI among PWE, could upregulate dopamine D_1_ receptors in the dentate gyrus and trigger epileptiform discharge (25). Although aging is inevitable, the progression of CI can be slowed by controlling metabolic syndrome and other neurological comorbidities. Detecting amyloid-β in people with late-onset epilepsy with unknown origin seems paramount.

Much can be done in terms of education and anxiety. We found that participants with no formal or primary education had worse cognitive function than those with a tertiary education, which was also supported by Lee et al. (26) who found that literacy activities alone could make a difference in cognition in PWE. However, due to stigma and overprotection from parents, many school-age children with epilepsy drop out of school. Hence, there is a need to strengthen advocacy for and education about epilepsy in society, and to encourage children with epilepsy to attend school long enough to complete at least primary education. Additionally, in our study, anxious symptoms rather than depressive symptoms were a risk factor for CI, which is consistent with the research by Miller et al. (6). This risk factor especially stood out among middle-aged PWE in our study. Some studies have reported that depressive symptoms are associated with cognitive aspects (27, 28); however, this conclusion was applicable only to elderly PWE in our study. Therefore, during the consultation, it is necessary for the physician to pay close attention to the mood of the patients to detect the symptoms according to ages. Moreover, the GAD-7 and NDDI-E can serve as a concise and time-saving scale in a clinical context for detecting chronic anxiety and depression. PWE receiving a professional but simple interpretation of the disease and encouraged to engage in communication with fellow epilepsy sufferers and to participate in social activities may be helped to relieve the mood disorders. CI caused by brain surgery and stroke was possibly associated with limbic system damages, and this might be the only risk factor of CI in young PWE before treatment. Moreover, moderate drinking and higher income were found here to be protective against CI, which might be due to these factors helping to relieve anxiety.

### Predictors of Cognitive Deterioration

At follow-up, the MoCA total scores had not changed significantly, indicating that the overall impact of anti-seizure medication treatment was mild. Moreover, the visuospatial and executive function and memory sub scores increased over the follow-up period. Consequently, we could reassure our patients that they can take anti-seizure medications without too many concerns about CI. For visuospatial and executive function, a more anxious status, and the use of high-risk treatments predicted functional decline. The former factor has been discussed above and the improvement of visuospatial and executive function could be partly explained by the improvement of anxious symptoms, and this improvement was evident in middle-aged PWE. The latter was found in a previous study on children and adolescents, i.e., anti-seizure medication treatment was found to preferentially affect attention and executive functions and adverse cognitive effects were found to differ between monotherapy and the use of at least three drugs (29). Executive functional deficits are prominent in neuropsychological side effects under topiramate, and attention and executive functions are always affected by anti-seizure medication (30). It might be the combined effect of multitherapy that slowed down the improvement of executive function. Jacob et al. (31) found that levetiracetam was associated with increased risk of dementia, but we found that only topiramate contributed, and only to declines in visuospatial and executive function. The overall effect of anti-seizure medication treatment on visuospatial and executive function was positive. As a result, it may be unnecessary for NDE patients to be excessively concerned about CI when using only one type of anti-seizure medication. Moreover, considering that risk is significant only with multiple-AED therapy and that no change was observed in the MoCA total score, it would not be reasonable to refuse single-AED therapy due to concerns about possible effects on single functions.

The objective memory improvement we observed could not be explained by age, changes in seizure frequency, use of high-risk treatments, depressive symptoms, or anxious symptoms, suggesting the presence of some underlying factor that we did not take into consideration. For the young PWE, the memory improvement was negatively affected by using treatments known to be high risk. Nevertheless, it seems that the overall effect of anti-seizure medication treatment on objective memory was positive, irrespective of the type and number of drugs, which is encouraging news for PWE taking these medicines.

High treatment risk was the only predictor of subjective memory decline in our study, which is similar to the findings of a previous study (21). The subjective memory decline we observed is therefore likely due to suggestion because people usually assume that multi-therapy has greater side effects than monotherapy and that the use of more than one type of anti-seizure medication implies severe disease and thus negative psychological implications. Moreover, it is common to explain to patients the possibility of cognitive decline caused by topiramate. All these factors may suggest that the patient's memory is deteriorating. Feldman et al. (32) found that subjective cognition was mainly associated with the number of anti-seizure medications, depressive symptoms, and seizure frequency. By comparing subjective memory between participants with refractory epilepsy and seizure-free, we found that refractory epilepsy was a predictor of subjective memory decline. However, we did not find that depression had a predictive role.

We did not find that seizure frequency played a role in CI. Previous studies have not found any adverse effect of a high seizure frequency on CI (33, 34). However, after 1 y of treatment, our participants with refractory epilepsy had worse cognitive performances than those who achieved freedom from seizures. Therefore, it can be concluded that refractory epilepsy is also a risk factor for cognitive decline, whether or not seizures directly affect cognition.

We did not find that duration of disease played a role in CI. A study on the relationship between the duration of temporal lobe epilepsy and cognitive abilities suggested that only a long duration (more than 30 y) was associated with cognitive impairment (35). The mean duration of epilepsy before anti-seizure medication treatment in our study was 3.7 y, which was relatively short and may explain why duration was not identified as a risk factor in our study.

PWE with TLE was found to have worse cognitive function than with FLE, especially in memory, and the memory test applied in our study was verbal memory. Some previous research on children supported the conclusion (36, 37). However, others have found commensurate memory impairment in PWE with TLE and with FLE (38). Further study should apply a complete battery test to verify it. After 1 y of anti-seizure medication treatment, picture-naming function improved in PWE with FLE in our study. Functional magnetic resonance imaging was used to locate the encephalic region and network connectivity responsible for naming, and temporal lobe was involved in it (39, 40). It might be the pathological changes in the temporal lobe for PWE with TLE leading to no improvement in picture-naming function to anti-seizure medication treatment.

### Limitations

(1) When evaluating cognition in cases of NDE before anti-seizure medication treatment, we did not simultaneously collect data on a control group, so that we could not fully confirm the cognitive improvement was caused by treatment. As our program is still going on and will continue for at least 2 y, the follow-up will be repeated every 6 months and we could collect the control group at the same time. (2) MoCA is a concise screening scale but the more detailed information of cognition could not be detected by it. Further study should administer a complete battery test to evaluate the CI. (3) When collecting the data on subjective memory, we used a brief but imprecise question, with the result that we could not categorically discriminate declines in subjective memory specifically from other forms of CI. Some scales designed for subjective memory could be applied in further study. (4) Comorbidities and concomitant therapies in elderly patients would be collected during the later follow-up and we would try to identify the genetic etiology in young patients.

## Conclusions

Almost half of the PWE analyzed had CI before starting an anti-seizure medication treatment. Aging, low educational level, an etiology of stroke or brain surgery, and anxious status were identified as risk factors for CI before anti-seizure medications use. Brain surgery for the young, anxious status for the middle-aged, and depressive status for the elderly were risk factors for CI at different ages. The elderly PWE had worse memory than the others. PWE with TLE had worse cognition, especially in memory and naming. The overall impact of anti-seizure medications on cognition was mild but positive. Refractory epilepsy was a predictor of cognitive decline. Subjective memory decline was predicted by using treatments known to be high risk and refractory epilepsy. Clarifying the risk factors may help physicians to interpret the probable risk of CI for each individual before starting anti-seizure medication treatment, which may lead to improved compliance.

## Data Availability Statement

The raw data supporting the conclusions of this article will be made available by the authors, without undue reservation.

## Ethics Statement

The studies involving human participants were reviewed and approved by the Ethics Committee of the First Hospital of Jilin University. Written informed consent to participate in this study was provided by the participants' legal guardian/next of kin.

## Author Contributions

NL was the first author and NL, JL, YC, XZ, RZ, ML, YL, and QZ were responsible for including participants, data entry, and following up. WL was the designer of this project. All authors contributed to the article and approved the submitted version.

## Conflict of Interest

The authors declare that the research was conducted in the absence of any commercial or financial relationships that could be construed as a potential conflict of interest.
